# Anticipated Pleasure and Displeasure for Future Social and nonsocial Events: A Scale Development Study

**DOI:** 10.1093/schizbullopen/sgad024

**Published:** 2023-08-22

**Authors:** Rui-Ting Zhang, Tian-xiao Yang, Zhao-ying Wang, Ming-yue Yang, Jia Huang, Ya Wang, Simon S Y Lui, Raymond C K Chan

**Affiliations:** Department of Psychology, Hunan Normal University, Changsha, China; Cognition and Human Behavior Key Laboratory of Hunan Province, Hunan Normal University, Changsha, China; Neuropsychology and Applied Cognitive Neuroscience Laboratory, Institute of Psychology, Chinese Academy of Sciences, Beijing, China; Department of Psychology, University of Chinese Academy of Sciences, Beijing, China; Department of Psychology, Hunan Normal University, Changsha, China; Cognition and Human Behavior Key Laboratory of Hunan Province, Hunan Normal University, Changsha, China; Department of Psychology, Hunan Normal University, Changsha, China; Cognition and Human Behavior Key Laboratory of Hunan Province, Hunan Normal University, Changsha, China; Neuropsychology and Applied Cognitive Neuroscience Laboratory, Institute of Psychology, Chinese Academy of Sciences, Beijing, China; Department of Psychology, University of Chinese Academy of Sciences, Beijing, China; Neuropsychology and Applied Cognitive Neuroscience Laboratory, Institute of Psychology, Chinese Academy of Sciences, Beijing, China; Department of Psychology, University of Chinese Academy of Sciences, Beijing, China; Department of Psychiatry, School of Clinical Medicine, The University of Hong Kong, Hong Kong Special Administrative Region, China; Neuropsychology and Applied Cognitive Neuroscience Laboratory, Institute of Psychology, Chinese Academy of Sciences, Beijing, China; Department of Psychology, University of Chinese Academy of Sciences, Beijing, China

**Keywords:** affective forecasting, anticipated pleasure, social, schizotypal traits, schizophrenia

## Abstract

**Background and Hypothesis:**

People with schizophrenia (SCZ) or schizotypal traits (ST) have difficulties in anticipating future pleasure and displeasure in social situations. However, no self-report scale has been developed to specifically capture these abilities. This study aimed to develop and validate the Social Affective Forecasting Scale (SAFS), and to examine how anticipated pleasure and displeasure are associated with ST and clinical symptoms in SCZ.

**Study Design:**

Study 1 recruited a main sample of 666 college students and a validation sample of 927 college students to complete the SAFS and other measurements for anhedonia. Exploratory factor analysis (EFA), confirmatory factor analysis (CFA), parallel analysis, and measurement invariance analysis were conducted. Study 2 recruited 2655 college students, 47 people with SCZ and 47 matched controls to complete the SAFS. Correlation analysis, regression analysis, and independent *t*-tests were performed.

**Study Results:**

Both EFA and CFA indicated a 4-factor model which was supported by parallel analysis in the validation sample. The SAFS showed good internal consistency, convergent validity, and strong invariance across sex. Interpersonal features of ST and negative symptoms in SCZ were associated with reduced anticipated pleasure for positive social events.

**Conclusions:**

The SAFS appears to be a reliable scale for evaluating anticipated pleasure and displeasure for future social and nonsocial events, and can be applied to study social anhedonia in individuals with ST and individuals with SCZ.

## Introduction

Anticipated emotion refers to an individual’s preconceived emotion before the occurrence of a future event.^1,2^ Accumulated evidence supports that anticipated emotion could direct future-oriented behaviors in everyday life,^3–5^ and contribute to anhedonia symptoms in both clinical and subclinical populations.^6–9^

Traditionally, anhedonia refers to the inability to experience pleasure.^10^ The “emotion paradox” in schizophrenia suggested that anhedonia can be parsed into consummatory pleasure (ie, the hedonic experience induced by the presentation of pleasurable cues) and anticipatory pleasure (ie, hedonic experience as an individual anticipates future pleasurable cues).^8^ Specifically, anticipatory pleasure includes (1) the prediction of future emotions (ie, anticipated pleasure; eg, how much will I enjoy the party with my friends?) and (2) “in-the-moment” pleasure experience evoked during such anticipation (eg, when I think about going hiking on Sunday, I get very excited).^8^ Research on anticipated pleasure can advance our knowledge on the mechanisms of anticipatory pleasure and anhedonia.

Anhedonia and altered anticipated pleasure are found in people with schizophrenia (SCZ) and schizotypal traits (ST).^11,12^ For instance, people with SCZ underestimated future pleasure but overestimated future displeasure compared with controls.^13^ Moreover, people with anhedonia may anticipate future pleasure differently in social vs. nonsocial aspects.^14–16^ For instance, people with SCZ anticipate less pleasure in social inclusion but not in social exclusion,^16^ and anticipate less pleasure than controls in social interaction conditions.^14^ Similar findings were observed in subclinical populations.^17,18^ For instance, Zhang et al^18^ found that people with negative ST anticipated less pleasure only in social conditions but not in nonsocial conditions, with detectable changes in functional connectivity.^18^

Empirical findings suggested that SCZ and individuals with ST showed abnormalities in anticipating displeasure.^19,20^ For instance, a daily diary study found that individuals with SCZ anticipated more negative emotions than they really experienced.^19^ Moreover, Martin et al^21^ found that individuals with SCZ anticipated more negative emotions than controls in a social interaction task.^21^ Likewise, people with negative ST anticipated more displeasure in social interaction.^20^ Taken together, the empirical evidence suggests that people with SCZ or ST exhibit difficulty in anticipating future positive and negative emotions, in particular for social rather than nonsocial events.

Previous studies often employed laboratory-based tasks to capture anticipated pleasure in social and nonsocial conditions.^17,18^ However, the laboratory-based tasks are usually time-consuming and less suitable for use as large-scale data collection. Complicated laboratory-based paradigms can be difficult for people with cognitive impairments. In contrast, self-report questionnaires are convenient and can measure everyday-life phenomena. While some studies adopted the Temporal Experience of Pleasure Scale (TEPS)^22^ and the Anticipatory and Consummatory Interpersonal Pleasure Scale (ACIPS)^23^ to measure hedonic experience for psychical and social conditions,^24,25^ these two self-reported scales did not directly measure anticipated pleasure, and the conditions they covered were only positive situations.

Therefore, a refined self-report measure for anticipated pleasure and displeasure in social and nonsocial conditions is needed. To address these issues, we conducted 2 related studies. In study 1, we aimed to develop and validate the “Social Affective Forecasting Scale (SAFS),” which would capture anticipated pleasure or displeasure for different types of events (ie, positive social, positive nonsocial, negative social, and negative nonsocial). In study 2, we examined the relationship between ST and anticipated pleasure and displeasure, and also evaluated patterns of anticipated pleasure and displeasure in people with SCZ, using the newly developed SAFS. We hypothesized that SAFS would demonstrate a 4-factor structure and acceptable psychometric properties. Moreover, we hypothesized that negative ST would be associated with reduced anticipated pleasure for positive social events, and increased anticipated displeasure for negative social events. In addition, we hypothesized that people with SCZ would exhibit social-specific impairments in anticipating future positive and negative events, compared with controls.

## Study 1

### Development of the SAFS

For item generation, we invited three professors in psychology who are experts in anhedonia, negative symptoms, prospection, and affective forecasting to form a focus group. Each professor provided 6–8 daily life events for positive social, positive nonsocial, negative social, and negative nonsocial conditions. In addition, we invited 20 college students and young adults from the community (see supplementary table 1 for their demographics) to propose daily events for each type of condition. Events with similar content or limited universality were excluded, resulting in an original item pool of 58 events.

We further invited another independent sample of 90 college students and young adults (see supplementary table 1 for their demographics) to evaluate the valence (“how happy this event is?”: 1 = very unhappy to 9 = very happy) and sociality (“how much social interactions are involved in this event?”: 1 = including no social interactions to 9 = including a lot of social interactions) of the 58 events in the original item pool. The average scores of valence and sociality for each event across 90 participants were calculated. Events were categorized into positive social, positive nonsocial, negative social, and negative nonsocial according to the scores of valence and sociality (See supplementary materials for details).

Following the aforementioned processes, we developed the original version of the 31-item SAFS (see supplementary table 2), which comprised 9 positive social items (eg, “attend best friend’s wedding/birthday party”), 8 positive nonsocial items (eg, “listen to your favorite music without being disturbed”), 8 negative social items (eg, “wait in a long line but someone rudely cut in lines”), and 6 negative nonsocial items (eg, “often wake up in the middle of the night and have nightmares”). Each item described a future event that may occur in daily life. Participants were required to imagine the event described in each item, and to anticipate how much pleasure they would feel as the event happens. Following the Circumplex Model of Affect proposed by Russell et al^26^ emotions can be measured in two dimensions (ie, valence and arousal), and the valence scale is a continuous spectrum from displeasure to pleasure.^26,27^ In line with previous literature,^28,29^ a bipolar scale from 1 (“very unhappy) to 7 (“very happy”) was used to capture the anticipated pleasure and displeasure. Therefore, for positive events, a higher score indicates that an individual predicts more anticipated pleasure; yet for negative events, a higher score indicates that an individual predicts less anticipated displeasure.

### Material and Methods

#### Participants

A total of 790 participants were recruited via an online advertisement. Participants completed the original version of SAFS, the TEPS, and the ACIPS. Four lie-detection items were incorporated into the scales to ensure the validity of participants’ responses.^30^ Detailed information regarding the lie-detection items are shown in the supplementary information. We excluded 124 participants who failed > 2 lie-detection items, similar to our early study.^30^ The final sample comprised 666 valid participants (female: 75.53%), with a mean age of 18.92 (*SD* = 1.18) and a mean education of 13.26 (*SD* = 0.98) years (see table 1). We used another independent sample to validate the factor structure. Specifically, following the same procedure, another 1190 participants were recruited, and 263 of them did not pass the lie-detection items, leaving 927 valid participants in the validation sample (female: 76.38%), with a mean age of 18.89 (*SD* = 0.83) and the mean education of 13.43 (*SD* = 0.66) years (see table 1).

**Table 1.** Demographic Information of the Main Sample and Validation Sample in Study 1

	Main Sample (*n* = 666)		Validation Sample (*n* = 927)	
	*Mean*	*SD*	*Mean*	*SD*
Sex (males: females)	163: 503		219: 708	
Age (years)	18.92	1.18	18.89	0.83
Length of Education (years)	13.26	0.98	13.43	0.66
TEPS_A	38.21	6.34	38.39	6.76
ACIPS_A	32.49	5.36	32.61	5.49

*Note:* TEPS_A, Temporal experience of pleasure scale anticipatory pleasure score; ACIPS_A, Anticipatory and consummatory interpersonal pleasure scale anticipatory pleasure score.

All participants provided informed written consent. They received 10 RMB (about 1.5 US dollars) as incentive after completion the questionnaires. This study was approved by the Research Ethics Committee of the Hunan Normal University.

#### Measurements

The TEPS^22^ was used to measure the anticipatory pleasure and consummatory pleasure. The ACIPS^23^ was used to assess the pleasurable experience in social conditions. Details of the TEPS and ACIPS are shown in supplementary information.

#### Data Analysis

The final sample (*N* = 666) was split into 2 sub-samples for the exploratory factor analysis (EFA) and the confirmatory factor analysis (CFA). First, we employed principal component analysis of EFA to examine the factor structure of SAFS. Given that the participants-to-item ratio for the EFA should be at least 10:1,^31^ we selected 310 participants (eg, participants whose ID belongs to 1–310 were selected) for the 31 items. The rest of the sample (*n* = 356) were used in the CFA to test the factor structure identified in the EFA. Before the EFA, the Kaiser–Mayer Olkin Measure of Sampling Adequacy (KMO)^32^ and Bartlett’s Test of Sphericity^33^ were conducted to test the feasibility of factor analysis given the dataset. EFA with promax rotation was performed, and all original items were included at first. Items cross-loaded on 2 or more factors were removed from the EFA sequentially, until no cross-loading items were found. Then, factors containing less than 3 items were deleted, and the EFA-identified model was generated. Next, CFA was carried out to test the hypothesized model and compare it with the EFA-identified model to find the best-fit model, using the maximum likelihood estimator. We therefore identify the best fit model for the final version of SAFS. The homogeneity reliability (ie, internal consistency) of the SAFS was examined using the Omega coefficient. The convergent validity was examined using correlations of the SAFS with the TEPS and the ACIPS. We applied Bonferroni corrections. To test the stability of the identified factor structure, the parallel analysis with 10 000 iterations was performed to determine the number of factors in an independent validation sample (*n* = 927). In addition, using the validation sample, multiple models (ie, configural invariance model, metric invariance model, and scalar invariance model) were tested sequentially to assess the measurement invariance (MI) between sex.

The EFA and the correlational analysis were performed using the Statistical Package for the Social Science (SPSS, version 22.0), and the CFA, the parallel analysis, and the MI analysis were conducted using Mplus.^34^

### Results

#### EFA of SAFS

Results of the KMO (KMO = 0.882) and Bartlett’s Test of Sphericity (*χ*^*2*^ = 4454.69, *df* = 465, *P* < .001) indicated that the dataset was appropriate for EFA. Upon performing the EFA, Items 30, 17, 20, 14, and 31 were removed since they were cross-loaded on two or more factors. Then, Items 18, 19, 21, and 22 were deleted since they were loaded on factors only contained 2 items. The scree plot and eigenvalue indicated a 4-factor model, and the factors accounted for 59.29% of the total variances.

Based on content of the items, the SAFS contained four factors, including the positive social events, the positive nonsocial events, the negative social events, and negative nonsocial events.

#### CFA of SAFS

Model fitting indices for the hypothesized model, the EFA-identified model, and the modified EFA-identified model are shown in table 2. We found that the EFA-identified model had better model-fitting indices than the hypothesized model. However, some indices (eg, CFI and RMSEA) in the EFA-identified model were not satisfactory. To improve the model fitting indices and to simplify the SAFS, we modified the EFA-identified model according to the modification index and removed those items having shared high variance (modification index >10).^35^ For example, Item 6 (“attend best friend’s wedding/birthday party”) and Item 7 (“go to the get-together with good friends or colleagues”) shared high variance, and the one in the pair having lower factor loading (ie, Item 7) was removed. Following the same procedure, Items 7, 8, 9, 10, and 16 (see supplementary table 2 for content of the items) were removed in the order of measurement error.^35^ The modified EFA-identified model included 17 items and four factors, with satisfactory indices. The factor loadings of the items in the best-fit model for the SAFS are shown in table 3.

**Table 2.** Model Fitting Indices for 3 Models of Social Affective Forecasting Scale in Study 1

	*Item count*	*Chi-square*	*df*	*AIC*	*BIC*	*RMSEA*	*SRMR*	*CFI*	*TLI*
Hypothesized model	31	1277.671	428	24580.418	24964.036	0.075	0.068	0.787	0.768
EFA-identified model	22	564.634	203	17469.664	17748.659	0.071	0.053	0.870	0.852
Modified EFA-identified model	17	223.998	113	13788.108	14008.979	0.053	0.044	0.937	0.924

*Note: n* = 356. EFA, exploratory factor analysis. SAFS, Social Affective Forecasting Scale

**Table 3.** Item Description and Factor Loading of Exploratory Factor Analysis of Social Affective Forecasting Scale in Study 1

Instruction: Please Read and Imagine the Described Event Happening to you in the Future, and Predict How You Will Feel When the Event Happens.	Factor Loadings			
	1	2	3	4
6. Attend best friend’s wedding/birthday party	0.875			
4. Receive blessings from friends	0.784			
5. Visit places with beautiful scenery with your family during vacation	0.722			
3. Offer prompt help when others need help	0.654			
1. Visit close friends who haven’t been in touch for a long time	0.645			
2. Meet people who have similar interests	0.606			
11. Listen to your favorite music without being disturbed		0.859		
12. Find an interesting book when you are home alone		0.762		
15. Find delicious food when you eat alone		0.747		
13. Relax yourself in nature and enjoy the fresh air		0.716		
28. A sudden power failure occurs when you are studying at home alone			0.802	
27. Hit your head accidently when you are home alone			0.729	
29. Forget to bring umbrellas on rainy days			0.690	
26. Often wake up in the middle of the night and have nightmares			0.493	
23. Wait in a long line but someone rudely cut in lines				0.842
24. Have unsatisfactory customer service from an online shopping platform				0.834
25. Be cheated of a sum of money by a scam group				0.657

*Note: n* = 310. factor 1, positive social events; factor 2, positive nonsocial events; factor 3, negative nonsocial events; factor 4, negative social events.

#### The Internal Consistency and Convergent Validity of SAFS

The final version of SAFS is shown in table 3. The final version of SAFS showed high internal consistency, with McDonald’s Omega values of 0.789 for the total score; 0.834 for the positive social subscale; 0.822 for the positive nonsocial subscale; 0.734 for the negative social subscale; and 0.692 for the negative nonsocial subscale.

The SAFS positive subscale scores were positively correlated with the TEPS anticipatory pleasure scores (positive social subscale: *r* = 0.41, *P* < .001; positive nonsocial subscale: *r* = 0.27, *P* < .001) and TEPS consummatory pleasure scores (positive social subscale: *r* = 0.35, *P* < .001; positive nonsocial subscale: *r* = 0.44, *P* < .001). Moreover, the SAFS positive subscale scores were positively correlated with the ACIPS anticipatory pleasure score (positive social subscale: *r* = 0.57, *P* < .001; positive nonsocial subscale: *r* = 0.24, *P* < .001) and the ACIPS consummatory pleasure score (positive social subscale: *r* = 0.56, *P* < .001; positive nonsocial subscale: *r* = 0.29, *P* < .001). The SAFS negative social subscale scores were negatively associated with the TEPS anticipatory pleasure scores (*r* = −0.13, *P* = .001). These correlations remained significant after Bonferroni's corrections. Other correlations did not survive Bonferroni corrections (*Ps* > .05). Detailed results could be found in supplementary table 3.

#### The Parallel Analysis and the MI of SAFS Across Sex

Based on the independent validation sample (*n* = 927), parallel analysis suggested that the SAFS contains four factors, and the factor structure showed consistency with the CFA-identified structure. For the MI analysis, the configural invariance model, the metric invariance model, and the scalar model provided a good fit to the data (see supplementary table 4). After applying metric constraints (ie, ΔCFI = −0.009; ΔRMSEA = 0.003; ΔSRMR = 0.010) and scalar constraints (ie, ΔCFI = −0.006; ΔRMSEA = 0.002; ΔSRMR = 0), the results showed only small changes in model fit which fell within the change criteria recommended by previous studies (ΔCFI < 0.01; ΔRMSEA < 0.015; ΔSRMR < 0.03).^36^

### Discussion

In Study 1, EFA and CFA indicated that the best fit model for SAFS has a 4-factor model, comprising the positive social, the positive nonsocial, the negative social, and the negative nonsocial factors. The 4-factor structure was supported by another independent sample using the parallel analysis. The SAFS also demonstrated good reliability, internal consistency, and convergent validity. Moreover, the MI for sex was established. In short, the newly developed SAFS is a reliable tool for evaluating anticipated pleasure and displeasure for different types of future events.

## Study 2

The newly developed SAFS was used to investigate the association between ST and anticipated pleasure and displeasure. Moreover, the SAFS was applied to evaluate the patterns of anticipated pleasure and displeasure in people with SCZ. In other words, we compared group differences (ie, SCZ group vs. controls) on the 4 factors of SAFS.

### Material and Methods

A total of 3506 young adults were recruited via an online advertisement. After excluding participants who were (1) younger than 18 years old (*n* = 111), (2) with missing data (*n* = 280), or (3) did not pass the lie-detection items (*n* = 460), we retained 2655 valid participants for final analysis. All participants completed the SAFS, TEPS, ACIPS, and the Schizotypal Personality Questionnaire (SPQ).^37^ Details of the SPQ are provided in the supplementary information.

Outpatients with SCZ (*n* = 47) as defined by the DSM-5^38^ were recruited from Guangji Hospital in Suzhou, China. Exclusion criteria included: (1) history of neurological disease, (2) history of head trauma, (3) substance abuse, and (4) IQ below 80. We recruited matched healthy people who did not have any diagnosable mental disorder (*n* = 47) from the nearby communities as controls. Clinical symptoms were assessed by the Positive and Negative Syndrome Scale (PANSS)^39^ and the Clinical Assessment Interview for Negative Symptoms (CAINS).^40^ Detailed information on the PANSS and the CAINS are shown in the supplementary information.

Regression analysis was used to explore the association between ST and scores on SAFS. Independent sample *t*-tests were performed to compare the group differences (ie, SCZ group versus controls) on the SAFS, the TEPS, and the ACIPS. Among SCZ participants, correlational analysis was conducted to evaluate the relationship between clinical symptoms and anticipated pleasure and displeasure.

This study was approved by the Research Ethics Committee of the Hunan Normal University. All participants provided informed written consent before joining this study.

### Results

As shown in table 4, the interpersonal features of the SPQ negatively predicted the SAFS positive social subscale scores (standardized *β* = −0.256, *P* < .001) and the positive nonsocial subscale scores (standardized *β* = −0.084, *P* = .001). Moreover, the cognitive-perceptual features of the SPQ positively predicted the SAFS positive social subscale score (standardized *β* = 0.062, *P* = .012) but negatively predicted the SAFS negative nonsocial subscale scores (standardized *β* = −0.081, *P* = .002). In addition, the disorganization features of the SPQ significantly predicted scores on the negative social subscale of the SAFS (standardized *β* = 0.056, *P* = .047).

**Table 4.** Regression Analysis on the Association Between Social Affective Forecasting Scale and Subdimensions of Schizotypal Traits in Study 2

Outcome Variables	Predictor	*R* ^*2*^	*Adjust R* ^*2*^	*F*	*Standardized Beta*	*P*
SAFS_PS	Cognitive-perceptual	0.071	0.069	67.066^***^	0.062	.012
	Interpersonal				−0.265	<.001
	Disorganization				−0.044	.106
SAFS_PN	Cognitive-perceptual	0.005	0.004	4.668^**^	0.026	.304
	Interpersonal				−0.084	.001
	Disorganization				0.003	.911
SAFS_NS	Cognitive-perceptual	0.003	0.001	2.318	−0.043	.091
	Interpersonal				−0.043	.095
	Disorganization				0.056	.047
SAFS_NN	Cognitive-perceptual	0.005	0.004	4.650**	−0.081	.002
	Interpersonal				−0.023	.372
	Disorganization				0.040	.160

*Note: n* = 2655; **P < .01; ***P < .001. SAFS, social affective forecasting scale; PS, positive social; PN, positive nonsocial; NS, negative social; NN, negative nonsocial.

The SCZ group and the control group were matched in sex, age, and length of education (*P*s > .05). The SCZ group reported reduced anticipated displeasure than controls on the SAFS negative social subscale (*P* = .027, *Cohen’s d* = 0.469). No significant group difference was found on positive social, positive nonsocial, or negative nonsocial subscales scores of the SAFS (table 5). Compared with controls, the SCZ group reported significantly lower scores on the TEPS anticipatory pleasure subscale (*P* = .039, *Cohen’s d* = 0.436), but similar scores on the TEPS consummatory pleasure subscale. No group difference was found on scores of the ACIPS.

**Table 5.** Comparisons of Demographic Information, and Ratings of the Social Affective Forecasting Scale, the Temporal Experience of Pleasure Scale and the Anticipatory and Consummatory Interpersonal Pleasure Scale Between SCZ Participants and Controls in Study 2

	SCZ (*N* = 47)		Control (*n* = 47)		*T/χ2*	*df*	*P*	*Cohen’s d*
	*Mean*	*SD*	*Mean*	*SD*				
Sex (male: female)	18:29		24:23		1.55	1	.300	
Age (years)	32.85	6.72	31.30	5.13	1.26	92	.211	0.263
Length of education (years)	13.94	2.31	14.49	1.97	−1.25	92	.214	0.261
SAFS_PS	35.68	4.29	36.98	3.72	−1.57	92	.121	0.327
SAFS_PN	23.38	3.35	23.06	3.27	0.47	92	.642	0.098
SAFS_NS	6.26	2.74	5.11	2.17	2.25	92	.027	0.469
SAFS_NN	10.38	3.42	11.04	2.93	−1.00	92	.319	0.209
TEPS_ A	35.26	5.34	37.66	5.78	−2.09	92	.039	0.436
TEPS_C	43.89	8.07	45.28	6.51	−0.91	92	.363	0.190
ACIPS_A	30.51	6.41	32.13	5.81	−1.28	92	.203	0.267
ACIPS_C	41.43	8.26	43.98	9.02	−1.43	92	.156	0.298

*Note:* SCZ, schizophrenia; SAFS, social affective forecasting scale; PS, positive social; PN, positive nonsocial; NS, negative social; NN, negative nonsocial; TEPS, temporal experience of pleasure scale; ACIPS, anticipatory and consummatory interpersonal pleasure scale; A, anticipatory pleasure scores; C, consummatory pleasure scores

As shown in supplementary table 6, the SAFS positive social subscale was negatively correlated with the PANSS negative symptoms subscale (*r* = −0.312, *P* = .043). Moreover, the SAFS positive social subscale was negatively correlated with the motivation and pleasure dimension of the CAINS (*r* = −0.322, *P* = .029).

### Discussion

In this study, we evaluated the relationship between ST and anticipated pleasure and displeasure using the SAFS. Moreover, we compared the group difference between SCZ and controls on anticipated pleasure and displeasure, using the SAFS.

Interpersonal features of the SPQ negatively predicted anticipated pleasure for future social and nonsocial events, and the predictive effect was stronger for social events (standardized *β* = −0.256, *P* < .001) rather than nonsocial events (standardized *β* = −0.084, *P* = .001). These results suggested that higher interpersonal features were associated with reduced anticipated pleasure for future positive events, especially social events. This finding is consistent with our hypothesis and other previous laboratory-based findings, which suggest that people with negative ST have difficulties in anticipating and experiencing pleasure (ie, reductions in anticipated and experienced pleasure), predominately in social conditions.^15,18^ Interestingly, we found that cognitive-perceptual features were associated with increased anticipated pleasure for positive social events, and increased anticipated displeasure for negative nonsocial events. Moreover, higher levels of disorganization features of the SPQ were found to be associated with reduced anticipated displeasure for negative social events. These findings suggest that different dimensions of ST demonstrate unique alterations in anticipating pleasure and displeasure for future events.

In consistent with our hypothesis, we found that positive social subscale was negatively associated with negative symptoms measured by the PANSS and the CAINS, indicating that higher levels of negative symptoms were associated with reduced anticipated pleasure for positive social events. However, contrary to our hypothesis, we found that SCZ patients anticipated less displeasure for future negative social events, compared with controls. Moreover, our results supported the “emotional paradox” by showing that SCZ patients demonstrated reduced anticipatory pleasure but intact consummatory pleasure compared with controls.

## General Discussion

In this investigation, we developed and validated the 17-item SAFS as a useful self-report scale to measure anticipated pleasure and displeasure for future social and nonsocial events. The SAFS demonstrated a 4-factor structure, capturing anticipated pleasure for future positive social, positive nonsocial events, and anticipated displeasure for future negative social and nonsocial events. The 4-factor structure was supported by a parallel analysis using an independent sample. MI of the 4-factor structure was demonstrated between sex. The SAFS scores were positively correlated with anticipatory pleasure as well as consummatory pleasure measured by the TEPS and the ACIPS. Moreover, using the SAFS, we explored the association between ST and anticipated pleasure and displeasure. In addition, we investigated the performance on anticipated pleasure and displeasure in people with SCZ. Taken together, the SAFS appears to have good convergent validity and discriminant validity.

Compared with other scales of anticipatory pleasure,^22,23^ the SAFS has several advantages. First, the SAFS is able to evaluate anticipated pleasure and displeasure specifically, contrary to other scales such as the TEPS and ACIPS which target mainly at the broader and less specific construct of anticipatory pleasure.^7^ Specifically, the anticipatory pleasure measured by the TEPS or the ACIPS included anticipated pleasure and other pleasure experiences, such as “in-the-moment” feelings during anticipation.^22,23^ Unlikely our refined scale of SAFS, the TEPS, and the ACIPS do not consider displeasure. The second important strength is that SAFS includes negative conditions which have been largely ignored by previous measurements for anhedonia, since recent literature has documented that individuals with SCZ show abnormalities when anticipating negative emotions,^20,21^ and a growing number of studies have pointed out the importance of considering both positive and negative events when investigating anhedonia.^18,21^ Therefore, the SAFS is useful for better understanding the relationship between anhedonia and anticipation of both positive and negative events. Third, the SAFS evaluates anticipated pleasure and displeasure in social and nonsocial conditions separately. To explain the “emotion paradox” of SCZ, the social-specific hedonic deficit theory proposed that anhedonia in SCZ may be restricted to social conditions.^15^ Previous studies using laboratory tasks also found that individuals with SCZ and ST showed social-specific impairments when forecasting future emotions.^17,18^ However, whether such altered anticipated pleasure and displeasure is specific to social domains remains unclear, and should be verified using large samples as well as tools with high ecological validity. The SAFS is a desirable tool, which is suitable for large data collection than laboratory-based tasks. As such, the development of the SAFS would benefit the understanding of social-specific deficits in hedonic experience in people with SCZ and ST. Taken together, the SAFS is a useful scale to advance our understanding of emotion since the SAFS can specifically capture anticipated pleasure and displeasure for various types of future events, which cannot be readily measured by many current measurement scales. More importantly, SAFS has a stable factor structure and can be used in future transdiagnostic research on the mechanisms of anhedonia.

Using the SAFS, we found negative ST was associated with reduced anticipated pleasure, predominately for social events, consistent with our hypothesis. This finding concurs with previous studies, which reported that people with negative ST anticipated less pleasure for social cues, instead of monetary cues.^17,41^ Taken together, our findings revealed social-specific deficits in anticipating positive emotions in people with ST, which is consistently observed in laboratory-based tasks and self-report scales. Given that anticipated pleasure is a cardinal component of anhedonia, the social-specific impairments in anticipated pleasure may explain why people with ST show anhedonia, especially for social cues.^15^

We found that cognitive-perceptual features were associated with increased anticipated pleasure for positive social events, and increased anticipated displeasure for negative nonsocial events. Previous study has revealed that positive ST was associated with bipolar-like experiences,^42^ and therefore people with positive ST may anticipate and experience more extreme emotions than controls. Indeed, previous literature reported that positive ST was associated with elevated anticipatory pleasure.^43^ Moreover, a recent study reported that, compared with controls, people with positive ST paid heightened attention to negative emotions,^44^ which may lead to increased anticipated displeasure. Taken together with previous findings, our results suggest that people with positive ST may anticipate pleasure and displeasure more extremely than controls. Unexpectedly, disorganization features were associated with reduced anticipated displeasure for negative nonsocial events. In a recent experience sampling methodology study, disorganized ST was associated with greater variability of negative affect.^45^ Therefore, the fluctuations of negative affect may affect the relationship between disorganization features and anticipated displeasure. However, future research is needed to verify this assumption. Our findings provided further evidence for the multidimensional model of ST, and found that different dimensions of ST were associated with distinct alterations in anticipating future emotions.

This current work also explored the performance of anticipated pleasure and displeasure in people with SCZ. We found that anticipated pleasure for positive social events was associated with more severe negative symptoms. Since the association between anticipated pleasure and negative symptoms was only found in social but not nonsocial events, such finding supported the social-specific hedonic deficit theory, which suggested that anhedonia in SCZ may be restricted to social conditions.^15^ Unexpectedly, people with SCZ anticipated less displeasure for future negative social events than controls, contrary to previous literature.^21^ However, some previous studies supported our findings and demonstrated that people with SCZ reported less ideal negative affect than controls.^46^ The heterogeneity of SCZ and different measures of negative affect may contribute to such inconsistent findings. In addition, our findings showed that people with SCZ reported reduced anticipatory pleasure than controls, but no significant group difference was found for consummatory pleasure. This finding supports the “emotion paradox” that people with SCZ demonstrate hedonic impairments in anticipating and retrieving pleasure, while having intact ability to experience “in-the-moment” pleasure.

Several limitations should be borne in mind. First, the original item pool was generated in the Chinese setting, and this may limit the generalizability of the SAFS to non-Chinese settings. Second, we did not examine the conceptual overlap between anticipated pleasure and anticipatory pleasure. Third, previous studies using ecological momentary assessments have indicated that environmental contexts play an influential role in the anhedonia and anticipatory pleasure; however, cross-sectional survey cannot assess the impact of environmental context.^47^ Future research may consider integrating the contents of the SAFS into ecological momentary assessments, to measure anticipated pleasure and displeasure in real-life setting and to evaluate whether anticipated pleasure and displeasure are contextually invariant. Lastly, we did not use any laboratory-based task to verify the results from the SAFS.

To conclude, the SAFS appears to be a reliable and valid self-report scale to capture anticipated pleasure and displeasure for future social and nonsocial events. Using the SAFS, we found that reduced anticipated pleasure for social events were associated with interpersonal features of ST, and negative symptoms of SCZ. Moreover, people with SCZ anticipated less displeasure for future social events compared with controls. Taken together, the SAFS is a suitable and promising tool to investigate social-specific hedonic deficits theory of anhedonia in people with SCZ.

## Supplementary Material

sgad024_suppl_Supplementary_Material
