# Supplementary material for: Anticipated Pleasure and Displeasure for Future Social and nonsocial Events: A Scale Development Study
Source: Schizophr Bull Open. 2023 Aug 22;4(1):sgad024. doi: 10.1093/schizbullopen/sgad024 (PMC11207892; doi:10.1093/schizbullopen/sgad024)
Supplement: sgad024_suppl_Supplementary_Material [file sgad024_suppl_Supplementary_Material.docx]

**Supplementary Materials**

Supplementary Table 1. Demographic information of participants for the scale development in Study 1

|  | Item development | |  | Ratings of 58 events | |
| --- | --- | --- | --- | --- | --- |
|  | college sample | society sample |  | college sample | society sample |
| Number | 10 | 10 |  | 56 | 34 |
| Sex (males: females) | 4:6 | 5:5 |  | 12:44 | 18:16 |
| Age (Mean±SD) | 18.50±5.31 | 30.70±1.43 |  | 19.20±1.78 | 32.00±9.56 |
| Years of education |  |  |  | 13.13±1.65 | 14.56±3.22 |

Supplementary Table 2. The original version of the SAFS

|  | Items |
| --- | --- |
| Positive social | 1. visit close friends who haven't been in touch for a long time |
|  | 2. meet people who have similar interests |
|  | 3. offer prompt help when others need help |
|  | 4. receive blessings from friends |
|  | 5. visit places with beautiful scenery with your family during vacation |
|  | 6. attend best friend’s wedding/birthday party |
|  | 7. go to the get-together with good friends or colleagues |
|  | 8. your lost wallet was returned by a kind stranger |
|  | 9. have a great time playing balls with your friends |
|  |  |
| Positive non-social | 10. take a relaxing walk after dinner |
|  | 11. listen to your favorite music without being disturbed |
|  | 12. find an interesting book |
|  | 13. relax yourself in nature and enjoy the fresh air |
|  | 14. have lazy mornings during holidays |
|  | 15. find delicious food when you eat alone |
|  | 16. make a delicious dish by yourself |
|  | 17. buy your desired gifts |
|  |  |
| Negative social | 18. be criticized by your teacher/boss for no reason |
|  | 19. your boss deliberately defaulted on wages |
|  | 20. your work is not understood and supported by your family |
|  | 21. have a big fight with your family and close friends |
|  | 22. dispute with others over important issues |
|  | 23. wait in a long line but someone rudely cut in lines |
|  | 24. have unsatisfactory customer service from an online shopping platform |
|  | 25. be cheated of a sum of money by a scam group |
|  |  |
| Negative non-social | 26. often wake up in the middle of the night and have nightmares |
|  | 27. hit your head accidently when you are home alone |
|  | 28. a sudden power failure occurs when you are studying at home alone |
|  | 29. forget to bring umbrellas on rainy days |
|  | 30. scores/grades were still not good no matter how hard you have tried |
|  | 31. your phone containing important information was broken and can not be fixed |

Supplementary Table 3. Correlations between SAFS, TEPS, and ACIPS in Study 1

|  | SAFS_PS | SAFS_PN | SAFS_NS | SAFS_NN | TEPS_A | TEPS_C | ACIPS_A | ACIPS_C |
| --- | --- | --- | --- | --- | --- | --- | --- | --- |
| SAFS_PS | 1 | .459^***^ | -.097^*^ | -0.003 | .407^***^ | .347^***^ | .569^***^ | .560^***^ |
| SAFS_PN |  | 1 | -0.021 | -0.027 | .269^***^ | .437^***^ | .243^***^ | .288^***^ |
| SAFS_NS |  |  | 1 | .348^***^ | -.131^***^ | -0.046 | -.081^*^ | -.089^*^ |
| SAFS_NN |  |  |  | 1 | -0.061 | 0.052 | -0.009 | -0.013 |
| TEPS_A |  |  |  |  | 1 | .571^***^ | .590^***^ | .616^***^ |
| TEPS_C |  |  |  |  |  | 1 | .465^***^ | .528^***^ |
| ACIPS_A |  |  |  |  |  |  | 1 | .845^***^ |
| ACIPS_C |  |  |  |  |  |  |  | 1 |

Note: n = 666; *: *p* < 0.05; ***: *p* < 0.001; SAFS = social affective forecasting scale; TEPS = temporal experience of pleasure scale; ACIPS = anticipatory and consummatory interpersonal pleasure scale; PS = positive social; PN = positive non-social; NS = negative social; NN = negative non-social; TEPS_A = anticipatory pleasure scores of temporal experience of pleasure scale; TEPS_C = consummatory pleasure scores of temporal experience of pleasure scale; ACIPS_A = anticipatory pleasure scores of anticipatory and consummatory interpersonal pleasure scale; ACIPS_C = consummatory pleasure scores of anticipatory and consummatory interpersonal pleasure scale.

Supplementary Table 4. Model comparison to determine measurement invariance by sex (Male [n = 219] vs Female [n=708]) in Study 1

|  | Goodness-of-fit | | | | | Model comparison | | | |
| --- | --- | --- | --- | --- | --- | --- | --- | --- | --- |
| Model | Chi-square | *df* | CFI | RMSEA | SRMR | Comparison | ΔCFI | ΔRMSEA | ΔSRMR |
| configural | 555.298 | 226 | 0.945 | 0.056 | 0.041 |  |  |  |  |
| metric | 620.256 | 239 | 0.936 | 0.059 | 0.051 | metric vs configural | -0.009 | 0.003 | 0.010 |
| scalar | 679.565 | 252 | 0.928 | 0.061 | 0.051 | scalar vs metric | -0.006 | 0.002 | 0.000 |

Note: n = 927; CFI = Comparative Fit Index; RMSEA = Root Mean Square Error of Approximation; SRMR = Standardized Root Mean Square Residual.

Supplementary Table 5. Gender effects on SAFS and anticipatory pleasure in Study 1

|  | Males  (n=163) | |  | Females  (n=503) | |  | *T* | *df* | *p* | *Cohen’s d* |
| --- | --- | --- | --- | --- | --- | --- | --- | --- | --- | --- |
|  | *Mean* | *SD* |  | *Mean* | *SD* |  |  |  |  |  |
| SAFS _PS | 36.60 | 4.79 |  | 37.99 | 3.44 |  | -3.42 | 218.72 | .001 | 0.309 |
| SAFS _PN | 23.91 | 3.61 |  | 24.81 | 2.73 |  | -2.94 | 225.04 | .004 | 0.265 |
| SAFS _NS | 5.42 | 2.05 |  | 5.24 | 1.83 |  | 1.01 | 251.42 | .312 | 0.091 |
| SAFS _NN | 12.06 | 2.82 |  | 11.70 | 2.92 |  | 1.40 | 664 | .162 | 0.126 |
| TEPS_A | 36.94 | 6.47 |  | 38.62 | 6.24 |  | -2.97 | 664 | .003 | 0.268 |
| TEPS_C | 44.71 | 8.02 |  | 45.77 | 7.26 |  | -1.57 | 664 | .116 | 0.142 |
| ACIPS_A | 31.53 | 5.57 |  | 32.79 | 5.26 |  | -2.62 | 664 | .009 | 0.236 |
| ACIPS_C | 44.12 | 8.25 |  | 45.81 | 7.91 |  | -2.30 | 265.20 | .022 | 0.208 |

*Note.* n=666. SAFS: social affective forecasting scale; PS: positive social subscale; PN: positive non-social subscale; NS: negative social subscale; NN: negative non-social subscale; TEPS_A = anticipatory pleasure scores of temporal experience of pleasure scale; TEPS_C = consummatory pleasure scores of temporal experience of pleasure scale; ACIPS_A = anticipatory pleasure scores of anticipatory and consummatory interpersonal pleasure scale; ACIPS_C = consummatory pleasure scores of anticipatory and consummatory interpersonal pleasure scale.

Supplementary Table 6. Correlations between the SAFS scores and clinical symptoms within schizophrenia patients in Study 2

|  | PANSS_P | PANSS-N | PANSS-G | CAINS_MAP | CAINS_EXP | SAFS_PS | SAFS_PN | SAFS_NS | SAFS_NN |
| --- | --- | --- | --- | --- | --- | --- | --- | --- | --- |
| PANSS_P | 1 | 0.043 | .484^**^ | -0.084 | -.323^*^ | 0.013 | 0.245 | -0.14 | 0.205 |
| PANSS_N |  | 1 | .363^*^ | .527^***^ | .367^*^ | -.321^*^ | -0.137 | 0.164 | -0.214 |
| PANSS_G |  |  | 1 | 0.154 | 0.15 | -0.242 | -0.091 | -0.059 | -0.076 |
| CAINS_MAP | |  |  | 1 | .292^*^ | -.322^*^ | -0.012 | 0.042 | 0.026 |
| CAINS_EXP |  |  |  |  | 1 | -0.152 | -0.164 | -0.141 | -0.212 |
| SAFS_PS |  |  |  |  |  | 1 | .638^***^ | -.422^**^ | -0.169 |
| SAFS_PN |  |  |  |  |  |  | 1 | -.423^**^ | -0.151 |
| SAFS_NS |  |  |  |  |  |  |  | 1 | .498^***^ |
| SAFS_NN |  |  |  |  |  |  |  |  | 1 |

*Note*: All schizophrenia patients completed the CAINS (n = 47) but only 40 schizophrenia patients completed the PANSS (n = 40). Correlational coefficient between PANSS and SAFS were based on 40 patients, while correlational coefficient between CAINS and SAFS were based on 47 patients. * = *p* < 0.05; ** = *p* < 0,01; *** = *p* <0.001. PANSS = Positive and Negative Syndrome Scale; P = positive subscale; N = negative subscale; G = general subscale; CAINS = Clinical Assessment Interview for Negative Symptoms; MAP = motivation and pleasure domain; EXP = expression domain; SAFS = social affective forecasting scale; PS = positive social; PN = positive non-social; NS = negative social; NN = negative non-social.

**Criteria for item selection of the original version**

Events were categorized into positive social, positive non-social, negative social, and negative non-social according to the scores of valence and sociality. An event would be categorized as “positive” if the valence rating was 0.5 *SD* above the mean (i.e., valence rating > 6.22), and “negative” if the valence was 0.5 *SD* below the mean (i.e., valence score < 4.18). The positive events were further classified into “positive social” events (i.e., the sociality score was 0.5 *SD* above the mean or > 6.69), and “positive non-social” events (i.e., the sociality score was 0.5 *SD* below the mean or < 5.75). Similarly, negative events were classified into “negative social” events if the sociality score was 0.5 *SD* above the mean (sociality score > 5.13), and “negative non-social” events if the sociality score was 0.5 *SD* below the mean (sociality score < 4.49). The means and *SDs* for sociality were calculated separately for positive events and negative events, because previous studies indicated higher sociality of positive events than negative events^1, 2^.

**Measurements**

***Temporal experience of pleasure scale (TEPS)***

The TEPS measures the anticipatory pleasure and consummatory pleasure^3^. The Chinese version of TEPS contains four factors, namely abstract anticipatory pleasure, contextual anticipatory pleasure, abstract consummatory pleasure, and contextual consummatory pleasure^4^. Each item is scored on a six-point Likert scale, with higher scores reflecting more pleasurable experience. The Chinese version of TEPS shows good psychometric properties^5^. The McDonald’s Omega of the TEPS in this study was 0.868.

***Anticipatory and consummatory interpersonal pleasure scale (ACIPS)***

The ACIPS assesses the pleasurable experience in social conditions^6^. It contains 7 items for anticipatory pleasure and 10 items for consummatory pleasure. Each item is scored on a six-point Likert scale, with higher scores indicating more pleasure experience. The Chinese version of ACIPS shows good psychometric properties^7^. The McDonald’s Omega of the ACIPS in this study was 0.917.

***Schizotypal Personality Questionnaire (SPQ)***

The SPQ captures the schizotypal traits in the non-clinical population. The SPQ comprises of three dimensions, namely the cognitive-perceptual, interpersonal and disorganization dimensions^8^. The SPQ has 74 items, each item describes psychotic-like experience and requires a yes/no answer. The total score of the SPQ ranges from 0 to 74, with higher scores indicating more schizotypal traits. The Chinese version of SPQ shows good psychometric features and has been applied in the Chinese sample^9^. The McDonald’s Omega of the ACIPS in this study was 0.959.

***The Positive and Negative Syndrome Scale (PANSS)***

The PANSS is used to evaluate the psychopathology of schizophrenia patients. The PANSS has three subscales, namely the positive, the negative, and the general symptom subscales, with higher scores indicating greater severity of symptoms ^10^. The PANSS showed good validity in previous research^10^.

***The Clinical Assessment Interview for Negative Symptoms (CAINS)***

The CAINSS is adopted to measure the negative symptoms of schizophrenia patients. The CAINS contains two factors, namely MAP (i.e., motivation and pleasure) and EXP (i.e., emotion expression), with higher scores indicating greater severity of negative symptoms^11^. The CAINS demonstrated good validity in clinical population^11^.

**Introduction of lie-detection items**

Four “Lie-detection items” were generated to exclude participants who were less concentrated when answering questionnaires. For example, item A (e.g., “I would rather be with people than alone”) and item B (e.g., “I would rather be alone”) are a pair of lie-detection item. Participants need answer YES or NO to each pair of lie-detection items, and lie-detection items would be scored if participants report inverse answers (e.g., answer both “YES” or “NO”) to a paired lie-detection item. Therefore, the score range of lie-detection items is from 0 to 4, with higher scores indicating less concentration.

**Reference**

**1.** Hur J, DeYoung KA, Islam S, Anderson AS, Barstead MG, Shackman AJ. Social context and the real-world consequences of social anxiety. *Psychological medicine* Sep 2020;50(12):1989-2000.

**2.** Kashdan TB, Collins RL. Social anxiety and the experience of positive emotion and anger in everyday life: an ecological momentary assessment approach. *Anxiety, stress, and coping* May 2010;23(3):259-272.

**3.** Gard DE, Gard MG, Kring AM, John OP. Anticipatory and consummatory components of the experience of pleasure: A scale development study. *Journal of Research in Personality* 2006;40:1086-1102.

**4.** Chan RC, Shi YF, Lai MK, Wang YN, Wang Y, Kring AM. The Temporal Experience of Pleasure Scale (TEPS): exploration and confirmation of factor structure in a healthy Chinese sample. *PLoS One* 2012;7(4):e35352.

**5.** Li Z, Lui SS, Geng FL, et al. Experiential pleasure deficits in different stages of schizophrenia. Schizophrenia research Aug 2015;166(1-3):98-103.

**6.** Gooding DC, Pflum MJ. The assessment of interpersonal pleasure: introduction of the Anticipatory and Consummatory Interpersonal Pleasure Scale (ACIPS) and preliminary findings. Psychiatry research Jan 30 2014;215(1):237-243.

**7.** Chan RC, Yang ZY, Li Z, Xie DJ, Gooding DC. Validation of the Chinese version of the Anticipatory and Consummatory Interpersonal Pleasure Scale. *PsyCh journal* Dec 2016;5(4):238-244.

**8.** Raine A. The SPQ: a scale for the assessment of schizotypal personality based on DSM-III-R criteria. *Schizophrenia bulletin* 1991;17(4):555-564.

**9.** Wang LL, Ma EPY, Lui SSY, Cheung EFC, Cheng KS, Chan RCK. Validation and extension of the Motivation and Pleasure Scale-Self Report (MAP-SR) across the schizophrenia spectrum in the Chinese context. *Asian journal of psychiatry* Mar 2020;49:101971.

**10.** Kay SR, Fiszbein A, Opler LA. The Positive and Negative Syndrome Scale (PANSS) for schizophrenia. *Schizophr Bull*. 1987;13(2):261–276.

**11.** Kring AM, Gur RE, Blanchard JJ, Horan WP, Reise SP. The Clinical Assessment Interview for Negative Symptoms (CAINS): final development and validation. *Am J Psychiatry*. 2013;170(2):165–172.
